# Sirolimus to the rescue: rapid therapeutic response in kaposiform hemangioendothelioma - case report and literature overview

**DOI:** 10.1186/s12887-026-07297-1

**Published:** 2026-07-08

**Authors:** Aleksandra Filipiuk, Łukasz Hutnik, Dominik Nguyen, Paweł Łaguna

**Affiliations:** 1 Department of Oncology, Pediatric Hematology, Clinical Transplantology and Pediatrics of the Children’s Clinical Hospital, Doctoral School, Medical University of Warsaw, Warsaw, Poland; 2 Department of Oncology, Pediatric Hematology, Clinical Transplantology and Pediatrics of the Children’s Clinical Hospital, Medical University of Warsaw, Warsaw, Poland; 3 Department of Pediatric Radiology, Medical University of Warsaw, Warsaw, Poland

**Keywords:** Kaposiform hemangioendothelioma, Sirolimus, Trough level, Biopsy

## Abstract

Kaposiform hemangioendothelioma (KHE) is a rare, locally aggressive vascular tumour of childhood. Due to its infiltrative growth and possible development of Kasabach-Merritt phenomenon (KMP) if untreated, early intervention is mandatory. Sirolimus, an mTOR (mammalian target of rapamycin) inhibitor, that has been used extensively in children following solid organ transplantation. Due to its pleiotropic effects, including the downregulation of angiogenic processes, immunomodulation, and reduction of platelet consumption, it has also shown promise in the management of vascular anomalies (VAs) in children.

We present our experience with a 7-month-old male patient with KHE of the lower limb and significant motor function impairment who was successfully treated with sirolimus.

This case provides evidence of the growing significance of the high efficacy of sirolimus in the treatment of vascular tumours. It also highlights the need for the development of standardized treatment guidelines for VAs in the paediatric population.

## Introduction

Kaposiform hemangioendothelioma (KHE) is a vascular tumour of intermediate malignancy [1]. The prevalence of KHE is approximately 0.91 per 100,000 children, with a slight male predominance. KHE manifests in infancy in 93% of cases, and in 60% of patients, onset occurs during neonatal period [2]. The most common locations are the extremities, followed by the trunk and the head-face-neck regions [2–4]. Visceral involvement- such as the thymus, mediastinum, and spleen - has been reported less frequently [5]. The clinical presentation of KHE is heterogenous and depends mainly on tumour location, size, and associated complications. Laboratory evaluation, including coagulation tests and a complete blood count, is essential in all suspected cases. Between 50% and 70% of patients develop.

Kasabach-Merritt phenomenon (KMP) - a potentially life-threatening consumptive coagulopathy with thrombocytopenia and hypofibrinogenemia - with a reported mortality of up to 30%. Thus, early identification and initiation of treatment are critical [2]. KHE is often misdiagnosed as other vascular tumours or malformations, including tufted angioma, infantile, congenital, verrucous malformation/haemangioma, spindle cell haemangioma, or Kaposi sarcoma, which may also occur in children [5].

While surgical resection was previously considered the definitive treatment, it is often not feasible due to the tumour’s infiltration across tissue planes and the associated risk of bleeding. Other treatment options have included vincristine, corticosteroids, propranolol, interferon, and radiotherapy. Since 2010, numerous reports have documented successful treatment with sirolimus. Most recently, Chinese experts published diagnostic and therapeutic guidelines for KHE [6]. However, these guidelines have not yet been extended to other countries [7].

We present a case of infantile KHE with complete remission following oral sirolimus therapy. This case underscores the efficacy of sirolimus and highlights the importance of a multidisciplinary approach in managing this complex and potentially life-threatening condition.

## Case presentation

A male infant with trisomy 21, from the gravida 4, para 3, was born at term following an uncomplicated spontaneous vaginal delivery, with a birth weight of 2,900 g and Apgar scores of 9–9–9–10. Shortly after birth, the infant experienced episodes of oxygen desaturation and respiratory distress. Echocardiography identified a perimembranous ventricular septal defect. Surgical repair of the cardiac defect was performed at four months of age. During the postoperative period, the patient developed pulmonary hypertension treated with inhaled nitric oxide. Subsequent echocardiographic follow-up revealed normal chamber dimensions and preserved biventricular systolic function.

**Fig. 1:**
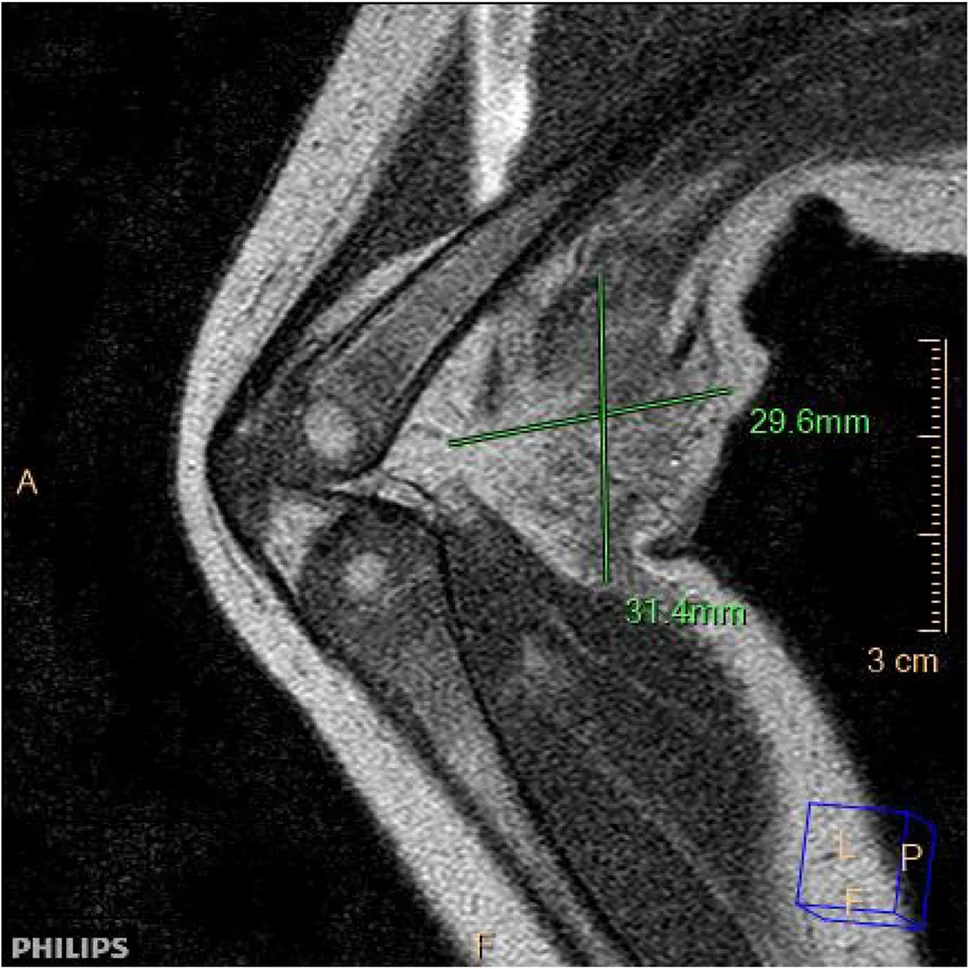
Coronal MRI view showing the patient’s lesion at the time of diagnosis

At seven months of age, the patient presented with a firm, immobile mass measuring approximately 40 × 35 mm in the left popliteal fossa, associated with overlying skin cyanosis. Additionally, a flexion contracture of the left knee exceeding 15 degrees was observed. Laboratory testing showed no haematological features suggestive of KMP. Ultrasound imaging identified an intratissue lesion with increased echogenicity and moderate internal vascularization. Magnetic resonance imaging (MRI) revealed a poorly defined lesion measuring 31.4 × 29.6 mm, involving the skin of the popliteal fossa, with infiltration into the semitendinosus and semimembranosus muscles. No evidence of bone involvement was noted (Fig.1). Histopathological analysis of a biopsy specimen confirmed the diagnosis of KHE, characterized by nodular vascular formations resembling capillary haemangiomas. Immunohistochemistry showed positivity for CD34, CD31, FL-1, ERG, and focal positivity for D2-40, with negativeGLUT-1 staining. The Ki-67 proliferation index was estimated at 1–2%. Overall, the findings were consistent with KHE involving the soft tissues of the left popliteal region, with infiltration of adjacent muscle structures, in the absence of bone involvement and without evidence of KMP at the time of diagnosis.

Sirolimus therapy was initiated at a dose of 0.8 mg/m²/dose twice daily, targeting trough levels of 5–15 ng/ml (Fig. 3). Simultaneously, the patient has been receiving ongoing physiotherapy as part of a multidisciplinary approach to care. Within two months of initiating therapy, ultrasound imaging revealed a marked reduction in lesion size (13 × 4 × 28 mm), accompanied by significant improvement in joint contracture. The child experienced no severe toxicity related to sirolimus. The therapy was well tolerated, with no severe adverse events. The only noted toxicity was transient grade 1 hypertriglyceridemia, classified according to Common Terminology Criteria for Adverse Events (CTCAE v5.0).

**Fig. 2:**
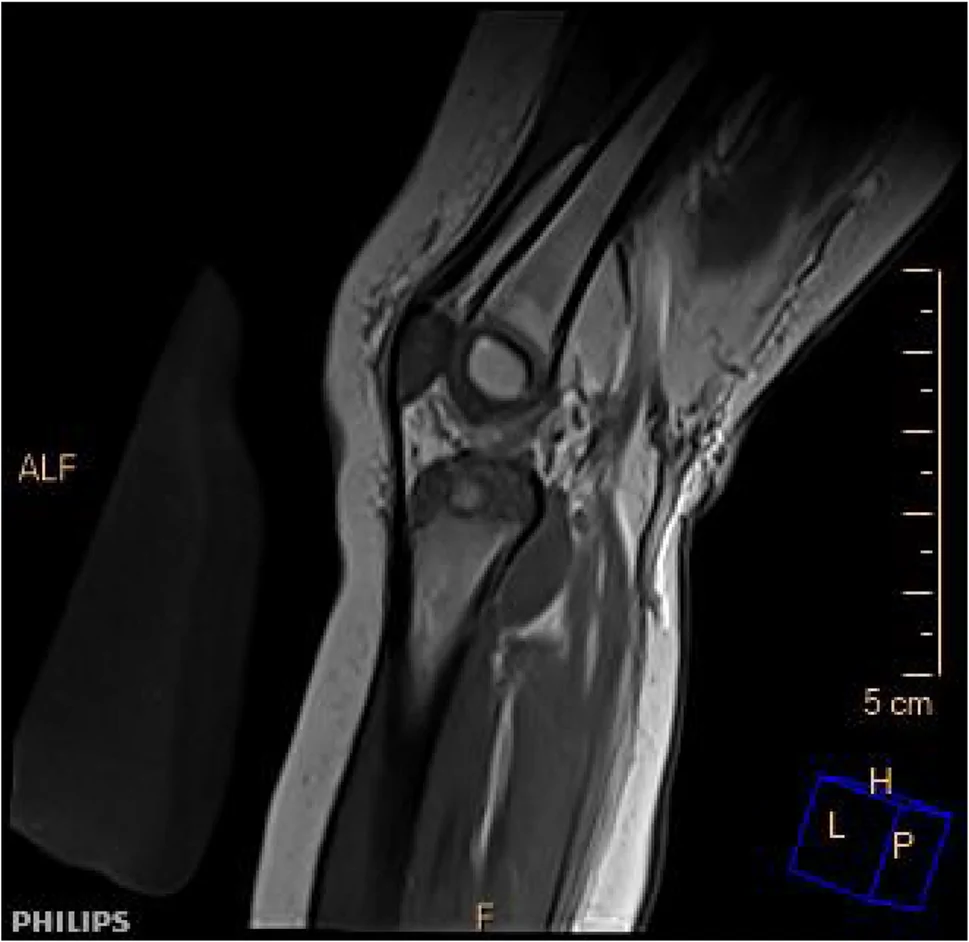
Coronal MRI scan indicating disease remission

MRI performed after four months of therapy confirmed complete remission (Fig. 2), and sirolimus therapy was successfully discontinued. Full knee mobility was restored. At 15 months of follow-up, the patient has been monitored clinically at three-month intervals, with no evidence of disease recurrence and sustained clinical well-being.

## Discussion

The International Society for the Study of Vascular Anomalies (ISSVA) provides an internationally accepted classification system for VAs, most recently updated in 2025. These are broadly divided into two categories: vascular tumours, which include proliferative changes of endothelial cells, and vascular malformations, which are structural vascular abnormalities lacking proliferative features [1].

KHE, initially introduced by Zukerberg et al. in 1993, is a locally aggressive/borderline vascular neoplasm [8]. The term reflects its morphological resemblance to Kaposi sarcoma (“kaposiform”) and its borderline malignant behaviour (“haemangioendothelioma”) [5]. KHE often infiltrates across tissue planes, involving the dermis, subcutaneous tissue, fascia, muscle, and occasionally bone.

In our patient, the diagnosis of KHE was confirmed through histopathological examination. Definitive diagnosis requires identification of infiltrative nodules composed of fascicles of spindle-shaped endothelial cells and slit-like vascular channels with irregular tumour margins. Immunohistochemical staining is supportive, with positive markers including D2-40, CD31, CD34, VEGFR-3, hyaluronan-1, and Prox-1, and negative staining for GLUT-1 [9].

Early diagnosis and prompt treatment are crucial for improving patient outcomes. Due to the broad clinical spectrum and lack of standardized treatment regimens, management of VAs is complex and necessitates a multidisciplinary team approach. This often includes paediatricians, haematologist-oncologists, radiologists, surgeons, pathologists, anaesthesiologists, and physiotherapists.

While surgical resection was considered the gold standard for definitive treatment, it is often not feasible due to the infiltrative nature of the lesion and risk of profuse bleeding. Moreover, treatment options have included high-dose steroids, chemotherapy (e.g., cyclophosphamide), antiplatelet therapy (e.g., aspirin), propranolol, embolization, radiotherapy, and sclerotherapy. In 2013, consensus treatment statements by Drolet et al. proposed a combination regimen of vincristine and corticosteroids for complicated KHE with KMP, and oral corticosteroids alone for KHE without KMP [10]. However, due to relevant side effects of corticosteroids and vincristine with prolonged or recurrent hospitalizations for treatment in neonates, inconsistent rates of complete remission, there has been a need for new, more target-specific therapeutic agents.

In recent years, sirolimus, used “off-label” in patients with VAs, has demonstrated increasing promise.

Sirolimus is a macrolide antibiotic originally isolated from *Streptomyces hygroscopicus* found in soil samples from Easter Island (Rapa Nui), hence the name “rapamycin”. It inhibits the mammalian target of rapamycin (mTOR), a serine-threonine kinase, suppressing cellular proliferation and lymphocyte activation by blocking the G1 phase of the cell cycle. Initially developed as an immunosuppressant for solid organ transplantation, sirolimus has since found applications in oncology and the treatment of VAs. A systematic review by Schmid et al. involving 215 patients with KHE concluded that sirolimus should be considered a first-line therapy for patients with KHE in treatment of KMP and tumour size reduction [4, 11]. At present, according to the KHE expert consensus panel in China, monotherapy with sirolimus is the preferred treatment for unresectable KHE. In patients who do not respond adequately to sirolimus, with or without corticosteroids, vincristine in combination with or without corticosteroids may be considered as a potential alternative [7]. Further support is provided by Wang et al., who reported that sirolimus is efficacious and well tolerated in patients with KHE [3]. On the other hand, results from a multicentre cohort study in the USA suggested that individualized treatment decisions depend on clinical scenario and patient/physician preferences. They observed no significant differences in therapy response or durability of disease comparing sirolimus- and vincristine-based regimens [12]. Prospective trials are still required to directly compare sirolimus monotherapy with other combined therapies.

**Fig. 3:**
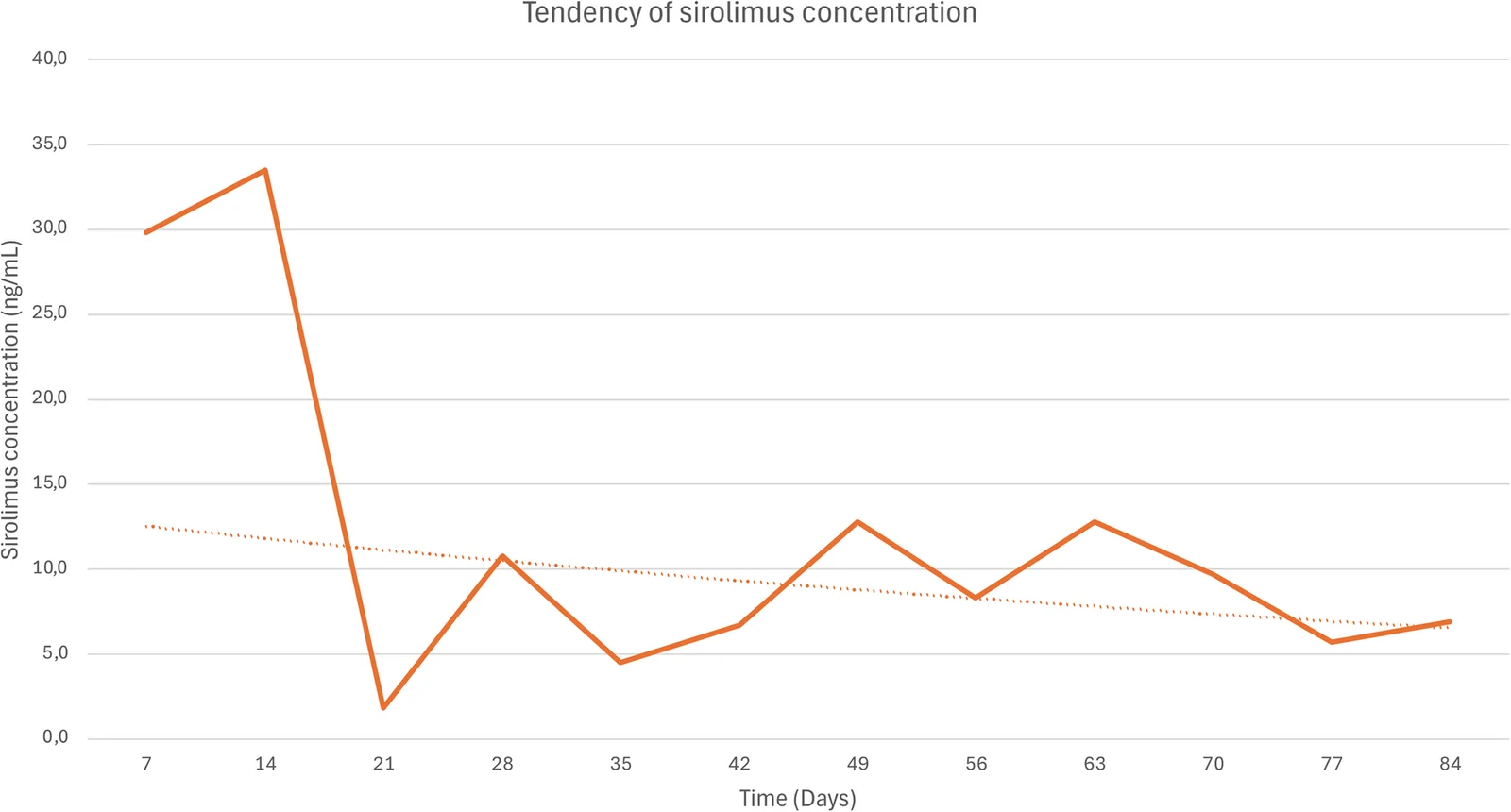
Sirolimus trough levels during therapy

Sirolimus oral formulation and favourable benefit-risk profile make it an attractive therapeutic option. Moreover, serum trough levels can be monitored, allowing for well-controlled therapy. Reported trough ranges typically fall between 5 and 15 ng/mL, with 10–15 ng/mL being the most targeted [3, 11]. However, lower trough levels (4–6 ng/mL) may be adequate for infants to improve safety [13]. In our patient, as reported by Wang et al. [3], the initial sirolimus plasma level was elevated but stabilized within the first month of therapy (Fig. 3).

When surgical biopsy is performed, the Chinese consensus statement recommends postponing initiation of sirolimus until the wound has healed, to avoid potential interference with tissue regeneration [7]. However, Mehl et al., in a retrospective cohort study, reported that sirolimus administration at lower concentrations (mean 6.5 ng/mL) may not significantly increase perioperative complication rates in paediatric patients undergoing resection of vascular anomalies [14]. These findings suggest that sirolimus concentration may be a potential variable influencing wound healing.

Overall, the safety profile of sirolimus is favourable [3]. In a study by Roessler et al., the most frequently observed severe adverse event (SAE) was viral pneumonia. The authors noted that some SAEs may be related to pre-existing immunodeficiency, for example, due to low immunoglobulin levels [15]. Similarly, Qiu et al. reported that the most common SAE leading to temporary treatment interruption was pneumonia, followed by upper respiratory tract infections, mucositis, and elevated liver transaminase levels. Interestingly, prophylactic use of trimethoprim-sulfamethoxazole did not reduce the incidence of infections [16]. Isolated cases of *Pneumocystis jirovecii* infection have been reported, primarily among patients receiving concurrent corticosteroid therapy [15]. More commonly, sirolimus is associated with transient dyslipidaemia, including hypertriglyceridemia and hypercholesterolemia, although these do not reach severe levels [17]. In our patient only mild, transient hypertriglyceridemia was observed, without the need for treatment interruption.

Importantly, the patient showed a rapid response to sirolimus, with marked tumour regression and restoration of motor function. Complete remission was achieved after a four-month course of therapy. At 15 months of post-withdrawal follow-up, no rebound tumour growth has been observed, with parallel improvement in psychomotor development.

## Conclusions

Due to the heterogeneous clinical presentation of KHE, no clear treatment guidelines currently exist, although rapid therapeutic intervention is often required to mitigate associated complications. Current evidence supports sirolimus as an effective, well-tolerated, and easily administered drug that may be considered a first-line option. However, optimal dosing and treatment duration remain to be defined. Therapy should ideally be managed in specialized centres with multidisciplinary expertise, and clinicians should remain vigilant for potential infectious and metabolic side effects.

## Data Availability

Data sharing is not applicable as no datasets were generated or analysed during the current study.

## Abbreviations

ISSVA: The International Society for the Study of Vascular Anomalies
KHE: Kaposiform hemangioendothelioma
KMP: Kasabach-Merritt phenomenon
MRI: Magnetic resonance imaging
mTOR: Mammalian target of rapamycin
SAE: Severe adverse effect
VA: Vascular anomaly
